# Hepatitis B Virus Reactivation and Mycobacterial Infections Associated With Ustekinumab: A Retrospective Study of an International Pharmacovigilance Database

**DOI:** 10.3389/fphar.2022.921084

**Published:** 2022-07-04

**Authors:** Jingjing Wang, Xiaozhen Geng, Xin Zhang, Yanfeng Xiao, Wenjun Wang

**Affiliations:** ^1^ Department of Pediatrics, Second Affiliated Hospital of Xi’an Jiaotong University, Xi’an, China; ^2^ Department of Infectious Diseases, Second Affiliated Hospital of Xi’an Jiaotong University, Xi’an, China

**Keywords:** ustekinumab, hepatitis B, tuberculosis, atypical mycobacterial infection, pharmacovigilance

## Abstract

**Background:** Reports were recently published on hepatitis B virus reactivation (HBVr), tuberculosis (TB), and atypical mycobacterial infection (AMI) in patients with ustekinumab treatment. However, the literature is limited to case reports and series. The study was aimed to investigate their relationships by using an extensive population-based database.

**Methods:** Using the United States Food and Drug Administration Adverse Event Reporting System (FAERS) database, we collected all cases of HBVr, TB, and AMI between 1 January 2009 and 30 September 2021, for ustekinumab and other drugs. Disproportionality was analyzed using the reporting odds ratio (ROR), which was considered significant when the lower limit of the 95% confidence interval (95% CI) was >1.

**Results:** Of the 18,760,438 adverse cases reported to FAERS for all drugs, 56,581 cases had been exposed to ustekinumab. Adverse events of HBVr, TB, and AMI were reported in 21, 210, and 20 cases, respectively. The ROR for HBVr with ustekinumab was 2.33 (95% CI, 1.52–3.58), for TB was 5.09 (95% CI, 4.44–5.84), and for AMI was 2.09 (95% CI, 1.35–3.24). In the ustekinumab exposure group, no death occurred in patients with HBVr, but one patient experienced life-threatening liver failure. For those with TB, 24 cases experienced hospitalization and 2 deaths occurred. No death occurred in patients with AMI but eight experienced hospitalization.

**Conclusion:** We identified positive signals between ustekinumab exposure and HBVr, TB, and AMI in FAERS. Although these complications are rare, clinicians using ustekinumab should be aware of the risks.

## Introduction

Ustekinumab is a humanized monoclonal antibody, which is approved for the treatment of plaque psoriasis, psoriatic arthritis, Crohn’s disease, and ulcerative colitis (FDA, 2020). Ustekinumab binds specifically to the p40 subunit shared by interleukin-12 (IL-12) and interleukin-23 (IL-23) (Luo et al., 2010). IL-12 and IL-23 are important pro-inflammatory cytokines that evoke immune and inflammatory responses, including natural killer cell activation, CD4^+^ T-cell differentiation and activation, the differentiation of CD4^+^ T cells toward the T-helper 1 phenotype, and induction of the T-helper 17 pathway (Benson et al., 2011; Teng et al., 2015; FDA, 2020).

The above IL-12/IL-23 pathways, which play roles in the pathogenesis of psoriasis, Crohn’s disease, and ulcerative colitis, however, are also important for host protection against bacterial, viral, parasitic, and fungal infections, especially for intracellular pathogens (Trinchieri, 2003; Langrish et al., 2004; Tato and Cua, 2008; Shen and Chen, 2018). Thus, the IL-12/IL-23 antagonist, ustekinumab, might increase the risk of infections, and reactivation of latent infections. Of these, hepatitis B virus (HBV) reactivation (HBVr) and tuberculosis (TB) are of particular concern. Since ustekinumab approval, cases of HBVr, TB, or atypical mycobacterial infection (AMI) have been occasionally reported in patients treated with ustekinumab (Sánchez-Moya and Daudén, 2012; Tsai et al., 2013; Errichetti and Piccirillo, 2014; Lynch et al., 2017; Shim et al., 2018; Renoux et al., 2020; Akiyama et al., 2021; Tominaga et al., 2021). As pre-marketing trials of ustekinumab usually excluded patients with chronic hepatitis B or active TB, or were conducted in populations with a low prevalence of exposure to HBV and TB, infection and reactivation events were rarely observed in clinical trials. Therefore, the associations of HBVr, TB, and AMI with ustekinumab are not clearly established.

In this study, we investigated the possible relationships between HBVr, TB, and AMI with ustekinumab use in clinical practice, using real-world pharmacovigilance data from the United States Food and Drug Administration Adverse Event Reporting System (FAERS).

## Materials and Methods

The FAERS is a post-marketing safety surveillance system for drugs and therapeutic biologic products (FAERS, 2021). It receives reports of adverse events (AEs) from healthcare professionals (e.g., physicians, pharmacists, nurses, and others), consumers (e.g., patients, family members, lawyers, and others), and manufacturers. Reporting is voluntary for healthcare professionals and consumers except manufacturers. The reports include information on patient demographics, medication use, AEs, indications, outcomes, and report sources. The FAERS database receives reports from both the United States and other countries and contained more than 23 million reports from 1968 to September 2021. The database allows for signal detection and quantification of an association between a drug and an adverse drug reaction. Our study obtained data from the FAERS public dashboard between the first quarter of 2009 and the third quarter of 2021 because ustekinumab was approved in 2009.

All AEs reported to FAERS were coded according to the Medical Dictionary for Regulatory Activities (MedDRA). For reports of HBV reactivation, we queried FAERS with the MedDRA term “Hepatitis B Reactivation.” For reports of TB, we queried using the following terms: “Adrenal Gland Tuberculosis,” “Bone Tuberculosis,” “Bovine Tuberculosis,” “Choroid Tubercles,” “Congenital Tuberculosis,” “Cutaneous Tuberculosis,” “Disseminated Tuberculosis,” “Ear Tuberculosis,” “Epididymitis Tuberculous,” “Extrapulmonary Tuberculosis,” “Female Genital Tract Tuberculosis,” “Intestinal Tuberculosis,” “Joint Tuberculosis,” “Lymph Node Tuberculosis,” “Male Genital Tract Tuberculosis,” “Meningitis Tuberculous,” “Esophageal Tuberculosis,” “Oral Tuberculosis,” “Pericarditis Tuberculous,” “Peritoneal Tuberculosis,” “Prostatitis Tuberculous,” “Pulmonary Tuberculoma,” “Pulmonary Tuberculosis,” “Renal Tuberculosis,” “Salpingitis Tuberculous,” “Spleen Tuberculosis,” “Thyroid Tuberculosis,” “Tuberculoma Of Central Nervous System,” “Tuberculosis,” “Tuberculosis Bladder,” “Tuberculosis Gastrointestinal,” “Tuberculosis Liver,” “Tuberculosis Of Central Nervous System,” “Tuberculosis Of Eye,” “Tuberculosis Of Genitourinary System,” “Tuberculosis Of Intrathoracic Lymph Nodes,” “Tuberculosis Of Peripheral Lymph Nodes,” “Tuberculosis Ureter,” “Tuberculous Abscess Central Nervous System,” “Tuberculous Laryngitis,” “Tuberculous Pleurisy,” and “Tuberculous Tenosynovitis.” For reports of AMI, we queried using the following terms: “Atypical Mycobacterial Infection,” “Atypical Mycobacterial Lower Respiratory Tract Infection,” “Atypical Mycobacterial Lymphadenitis,” “Atypical Mycobacterial Pneumonia,” “Atypical Mycobacterium Pericarditis,” “Disseminated *Mycobacterium Avium* Complex Infection,” “*Mycobacterium Abscessus* Infection,” “*Mycobacterium Avium* Complex Infection,” “*Mycobacterium Chelonae* Infection,” “*Mycobacterium Fortuitum* Infection,” “*Mycobacterium Hemophilum* Infection,” “*Mycobacterium Kansasii* Infection,” “*Mycobacterium Marinum* Infection,” and “*Mycobacterium Ulcerans* Infection.”

HBVr, TB, and AMI cases among patients treated with ustekinumab were respectively compared to all HBVr, TB, and AMI events reported in the database due to other drugs. When available, the following clinical characteristics of reported cases were also collected and analyzed: sex, age, reporter type, report countries, indications, concomitant product names, and reaction outcomes. Duplicate reports were removed according to the unique case ID and the case characteristics. Cases were compiled into Microsoft Excel 2019.

Disproportionality signal analyses were performed by calculating the reporting odds ratio (ROR) with its 95% confidence interval (CI) (Supplementary Material S1) (Stricker and Tijssen, 1992; van der Heijden et al., 2002; van Puijenbroek et al., 2002; Rothman et al., 2004). The ROR is defined as the ratio of two odds. The numerator consists of the odds of the number of reports of interested AE (HBVr, TB, or AMI) to the index drug ustekinumab and to other drugs. The denominator consists of the odds of the number of reports of other AEs to the index drug ustekinumab and to other drugs (Supplementary Material S1). For example, if the value of ROR is x for HBVr with ustekinumab, it means the odds of reporting HBVr with ustekinumab use is x times of reporting the AE with other medications use in FAERS. When the lower limit of the 95% CI of the ROR was >1 with at least three cases (Antonazzo et al., 2019; Sardella and Lungu, 2019), the ROR was considered significant. The likelihood of associations between ustekinumab with HBVr, TB, and AMI were assessed using the two-sided chi-square or Fisher’s exact tests, as warranted. All analyses were conducted using Stata/SE 12.0 (StataCorp LP, College Station, TX, United States) and statistical significance was defined as *p* < 0.05. Institutional review board approval was not required because the FAERS is open to the public and patient records are anonymized and deidentified. Our study adhered to the Guidelines for Accurate and Transparent Health Estimates Reporting (GATHER) statement (Supplementary Material S2) (Stevens et al., 2016).

## Results

There were a total of 18,760,438 AE reports between 1 January 2009 and 30 September 2021 in FAERS, of which 56,581 (0.30%) individual cases were related to ustekinumab. Indication for use was reported in 43,474 cases (76.83%), with 26,509 (46.85%) for psoriasis/psoriatic arthritis, 15,005 (26.52%) for Crohn’s disease/ulcerative colitis, and 1,960 (3.46%) for other conditions. Country of AE origin was reported in 55,918 cases (98.83%), including 40,431 (71.46%) from the Americas, 12,493 (22.08%) from Europe, 1,720 (3.04%) from Asia, 1,234 (2.18%) from Oceania, and 40 (0.07%) from Africa. Most reported AEs related to ustekinumab were serious (61.31%). Deaths occurred in 1,269 (2.24%) patients with available follow-up (Table 1).

**TABLE 1:** Characteristics of patients with HBVr, TB, and AMI related to ustekinumab between 1 January 2009 and 30 September 2021 in FAERS.

	Total (%)	HBVr (%)	TB (%)	AMI (%)
Number of cases	56,581	21	210	20
Areas				
Americas	40,431 (71.46)	4 (19.05)	90 (42.86)	10 (50.00)
Europe	12,493 (22.08)	3 (14.29)	84 (40.00)	2 (10.00)
Asia	1,720 (3.04)	14 (66.67)	32 (15.24)	8 (40.00)
Oceania	1,234 (2.18)	0 (0)	2 (0.95)	0 (0)
Africa	40 (0.07)	0 (0)	2 (0.95)	0 (0)
Unspecified	663 (1.17)	0 (0)	0 (0)	0 (0)
Indication				
Psoriasis/psoriatic arthritis	26,509 (46.85)	20 (95.24)	138 (65.71)	13 (65.00)
Crohn’s disease/ulcerative colitis	15,005 (26.52)	0 (0)	32 (15.24)	5 (25.00)
Others	1,960 (3.46)	0 (0)	5 (2.38)	2 (10.00)
Unknown	13,107 (23.17)	1 (4.76)	35 (16.67)	0 (0)
Sex				
Male	22,554 (39.86)	12 (57.14)	104 (49.52)	8 (40.00)
Female	29,049 (51.34)	5 (23.81)	68 (32.38)	8 (40.00)
Unspecified	4,978 (8.80)	4 (19.05)	38 (18.10)	4 (20.00)
Median age, years (IQR)	50 (38–61), *n* = 33,176	47 (41–50), *n* = 13	50 (37–64), *n* = 97	59 (53–66), *n* = 13
Type of reactions				
Serious	34,687 (61.31)	19 (90.48)	206 (98.10)	20 (100)
Non-serious	21,894 (38.69)	2 (9.52)	4 (1.90)	0 (0)
Concomitant medications				
No	49,427 (87.36)	17 (80.95)	181 (86.19)	14 (70.00)
Yes	7,154 (12.64)	4 (19.05)	29 (13.81)	6 (30.00)
Other reactions				
No		14 (66.67)	132 (62.86)	7 (35.00)
1 other reaction		2 (9.52)	35 (16.66)	2 (10.00)
2 or more reactions		5 (23.81)	43 (20.48)	11 (55.00)
Outcome^#^				
Died	1,269 (2.24)	0 (0)	2 (0.95)	0 (0)
Life threatening	1,045 (1.85)	1 (4.76)	2 (0.95)	0 (0)
Hospitalized	11,516 (20.35)	1 (4.76)	24 (11.43)	8 (40.00)
Disabled	894 (1.58)	0 (0)	0 (0)	2 (10.00)
Others	24,687 (43.63)	18 (85.71)	195 (92.86)	19 (95.00)
Non-serious	21,894 (38.69)	2 (9.52)	4 (1.90)	0 (0)
Reporter				
Health care professional	36,952 (65.31)	18 (85.71)	145 (69.05)	18 (90.00)
Consumer	18,910 (33.42)	3 (14.29)	64 (30.48)	2 (10.00)
Unspecified	719 (1.27)	0 (0)	1 (0.48)	0 (0)

*Cases with serious reactions could have one or more of the following outcomes: died, life threatening, hospitalized, disabled, and others. AMI, atypical mycobacterial infection; FAERS, United States Food and Drug Administration Adverse Event Reporting System; HBVr: hepatitis B virus reactivation; IQR, interquartile range; TB, tuberculosis.

**FIGURE 1:**
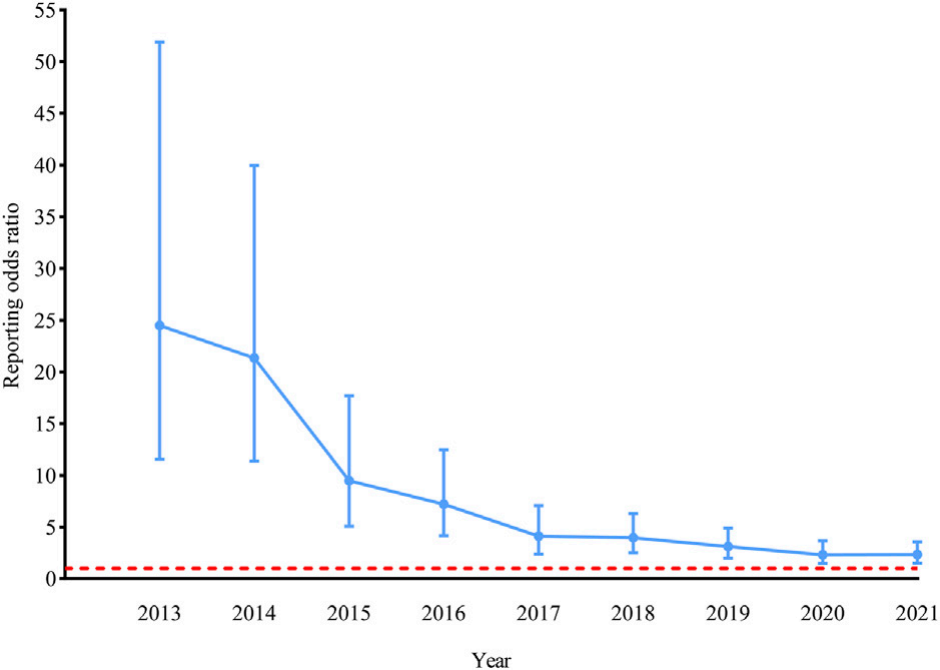
| Reporting odds ratio (ROR) for hepatitis B virus reactivation with ustekinumab. The ROR was not calculated before 2013 because there were less than three cases of hepatitis B virus reactivation reported with ustekinumab in the United States Food and Drug Administration Adverse Event Reporting System (FAERS). Data was updated to 30 September 2021. The bars represent the 95% confidence intervals of the ROR. The red line represents the threshold for signal detection (ROR = 1).

Twenty-one cases were reported for HBVr with ustekinumab use, compared to 2,983 cases by using other medications (Table 2). The ROR was 2.33 (*p* < 0.001, 95% CI, 1.52–3.58). The evolution of ROR over time showed that the disproportionality remained significant for the association of ustekinumab and HBVr (Figure 1). Stratified analyses by sex, age, reporter, and reporter region showed that the ROR was elevated in each stratum, but only statistically significant in the strata of male, aged less than 65 years, consumer reporter, and reported from US (Supplementary Table S1 in Supplementary Material S3). Most cases were from Asia (66.67%). Twelve (57.14%) were males and five (23.81%) were females, whereas in four (19.05%) cases, sex was not specified. In 17 cases with HBVr, ustekinumab was the only suspected drug. In the remaining four cases, concomitant drugs were also suspected for causing AEs. These drugs included methotrexate, adalimumab, cyclosporine, apremilast, and prednisone. Fourteen (66.67%) cases had only HBVr, while seven (33.33%) cases had one or more additional reactions including hepatobiliary (4 cases), gastrointestinal (3 cases), other infectious (2 cases), pulmonary (1 case), hematological (1 case), neurological (1 case), malignant (1 case), and other (3 cases) complications. In the ustekinumab exposure group, no death occurred in patients with HBVr, but one patient experienced life-threatening acute liver failure.

**TABLE 2:** Hepatitis B virus reactivation related to ustekinumab and other medications between 1 January 2009 and 30 September 2021 in FAERS.

	Ustekinumab	All other medicines	Sum	ROR (95%CI)	*p* value
Hepatitis B virus reactivation	21	2,983	3,004	2.33 (1.52–3.58)	<0.001
All other events	56,560	18,700,874	18,757,434		
Sum	56,581	18,703,857	18,760,438		

CI, confidence interval; FAERS, United States Food and Drug Administration Adverse Event Reporting System; ROR, reporting odds ratio.

**FIGURE 2:**
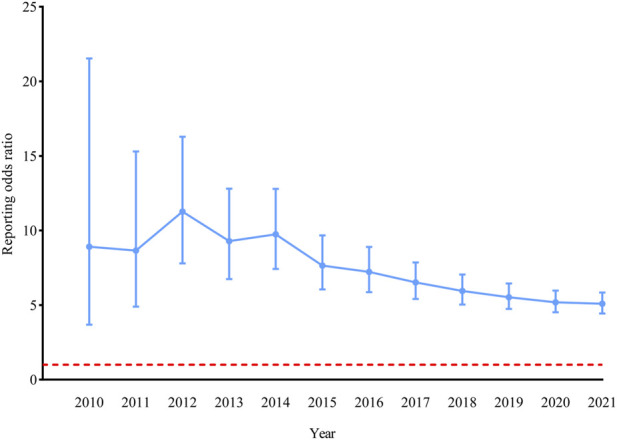
| Reporting odds ratio (ROR) for tuberculosis with ustekinumab. The ROR was not calculated before 2010 because there were less than three cases of tuberculosis reported with ustekinumab in the United States Food and Drug Administration Adverse Event Reporting System (FAERS). Data was updated to 30 September 2021. The bars represent the 95% confidence intervals of the ROR. The red line represents the threshold for signal detection (ROR = 1).

TB was noted in 210 cases using ustekinumab compared with 13,671 cases using other medications (Table 3). The ROR was 5.09 (*p* < 0.001, 95% CI, 4.44–5.84). The evolution of ROR over time showed that the disproportionality remained significant for the association of ustekinumab and TB (Figure 2). Stratified analyses by sex, age, reporter, and reporter region showed that the ROR was significantly elevated in each stratum (Supplementary Table S2 in Supplementary Material S3). Most cases (80.95%) lacked the report of site of TB infection. In the remaining 40 cases with report of infection site, 17 had pulmonary TB, 17 had extrapulmonary TB, and 8 had disseminated TB. Lymph nodes were the most involved extrapulmonary site (9 cases), followed by peritoneum (2 cases), bone (2 cases), pleura (1 case), pericardium (1 case), meninges (1 case), liver (1 case), and spleen (1 case). Most cases were from the Americas (42.86%) and Europe (40.00%). There were 104 (49.52%) males and 68 (32.38%) females, whereas in 38 (18.10%) cases, sex was not specified. In 181 of 210 cases with TB, ustekinumab was the only suspected drug. In the remaining 29 cases, concomitant drugs were also suspected for causing AEs. Adalimumab, infliximab, etanercept, and secukinumab were the most commonly used drugs concomitantly. In 78 of 210 patients with TB due to ustekinumab, additional AEs were reported including other infections (22 cases), cardiac (11 cases), dermatological (11 cases), gastrointestinal (9 cases), neurological (9 cases), hematological (7 cases), malignant (7 cases), vascular (6 cases), renal and urinary (6 cases), hepatobiliary (5 cases), musculoskeletal (5 cases), pulmonary (4 cases), immunological (4 cases), psychological (3 cases), ophthalmic (3 cases), infusion-related (2 cases), and other complications (52 cases). In the ustekinumab exposure group, 24 patients experienced hospitalization and 2 deaths occurred in patients with TB.

**TABLE 3:** Tuberculosis related to ustekinumab and other medications between 1 January 2009 and 30 September 2021 in FAERS.

	Ustekinumab	All other medicines	Sum	ROR (95%CI)	*p* value
Tuberculosis	210	13,671	13,881	5.09 (4.44–5.84)	<0.001
All other events	56,371	18,690,186	18,746,557		
Sum	56,581	18,703,857	18,760,438		

CI, confidence interval; FAERS, United States Food and Drug Administration Adverse Event Reporting System; ROR, reporting odds ratio.

**FIGURE 3:**
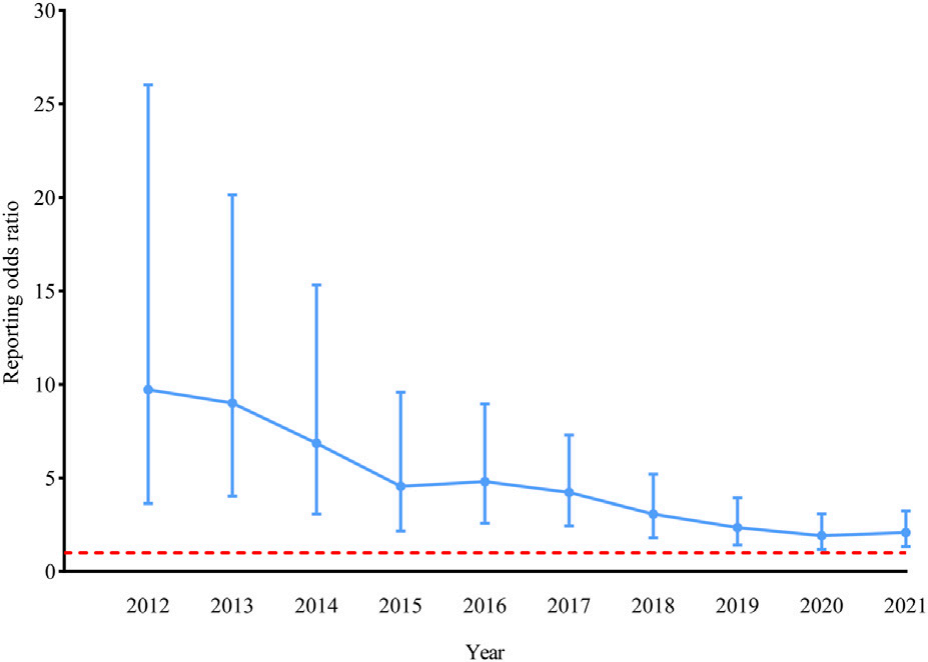
| Reporting odds ratio (ROR) for atypical mycobacterial infection with ustekinumab. The ROR was not calculated before 2012 because there were less than three cases of atypical mycobacterial infection reported with ustekinumab in the United States Food and Drug Administration Adverse Event Reporting System (FAERS). Data was updated to 30 September 2021. The bars represent the 95% confidence intervals of the ROR. The red line represents the threshold for signal detection (ROR = 1).

AMI was noted in 20 cases using ustekinumab compared with 3,164 cases using other medications (Table 4). The ROR was 2.09 (*p* = 0.001, 95% CI, 1.35–3.24). The evolution of ROR over time showed that the disproportionality remained significant for the association of ustekinumab and AMI (Figure 3). Stratified analyses by sex, age, reporter, and reporter region showed that the ROR was elevated in each stratum, but only statistically significant in the stratum of aged 65 years or more (Supplementary Table S3 in Supplementary Material S3). Eight patients did not report the species of AMI. In the remaining 12 cases, 5 were infected with *Mycobacterium avium complex*, 3 with *M. fortuitum*, 2 with *M. abscessus*, and 2 with *M. marinum*. Most cases were from the Americas (50.00%) and Asia (40.00%). Males and females were eight cases each, whereas in four cases, sex was not specified. In 14 cases, ustekinumab was the only suspected drug. In the remaining six cases, concomitant drugs were also suspected for causing AEs. Infliximab, golimumab, and prednisolone were the most commonly used drugs concomitantly. In 13 of 20 patients who had AMI due to ustekinumab, additional AEs were reported including other infections (6 cases), musculoskeletal (6 cases), neurological (4 cases), dermatological (4 cases), infusion-related (3 cases), immunological (3 cases), pulmonary (2 cases), gastrointestinal (2 cases), ophthalmic (2 cases), cardiac (1 cases), haematological (1 cases), malignant (1 cases), vascular (1 cases), and other complications (8 cases). In the ustekinumab exposure group, no death occurred in patients with AMI, but eight patients experienced hospitalization, including two disabled.

**TABLE 4:** Atypical mycobacterial infection related to ustekinumab and other medications between 1 January 2009 and 30 September 2021 in FAERS.

	Ustekinumab	All other medicines	Sum	ROR (95%CI)	*p* value
Atypical mycobacterial infection	20	3,164	3,184	2.09 (1.35–3.24)	0.001
All other events	56,561	18,700,693	18,757,254		
Sum	56,581	18,703,857	18,760,438		

CI, confidence interval; FAERS, United States Food and Drug Administration Adverse Event Reporting System; ROR, reporting odds ratio.

## Discussion

Ustekinumab, as a first-in-class agent targeting the IL-12/23 pathway, was approved by the United States Food and Drug Administration in 2009 to treat moderate-to-severe plaque psoriasis in patients who are candidates for phototherapy or systemic therapy (FDA, 2020). Compared with placebo or etanercept, a tumor necrosis factor (TNF) inhibitor, it effectively reduces the disease activity and provides sustained improvements in both clinical outcomes and health-related quality of life (Leonardi et al., 2008; Papp et al., 2008; Griffiths et al., 2010). As trials have demonstrated its efficacy in psoriatic arthritis, and more importantly in those previously exposed to TNF inhibitors, it was also approved for active psoriatic arthritis, alone or in combination with methotrexate (McInnes et al., 2013; Kavanaugh et al., 2014; Ritchlin et al., 2014). Subsequently, it was further approved for other immunological diseases, including Crohn’s disease and ulcerative colitis, especially for those who have failed to respond or are intolerant to either conventional therapy or anti-TNF therapy, or have medical contraindications to such therapies (Feagan et al., 2016; Sands et al., 2019). The successful off-label use of ustekinumab has been reported in giant cell arteritis, Behçet disease, systemic lupus erythematosus, and hidradenitis suppurativa (Conway et al., 2016; Maarouf et al., 2018; van Vollenhoven et al., 2018; London et al., 2021).

Due to the roles of the IL-12/IL-23 pathway in protecting the host from pathogens, however, ustekinumab may result in unintended downstream consequences such as the development of new infections or reactivation of underlying infections (Trinchieri, 2003; Langrish et al., 2004; Tato and Cua, 2008; Shen and Chen, 2018). Since ustekinumab approval, HBVr, TB, and AMI have been reported occasionally in patients treated with it (Sánchez-Moya and Daudén, 2012; Tsai et al., 2013; Errichetti and Piccirillo, 2014; Lynch et al., 2017; Shim et al., 2018; Renoux et al., 2020; Akiyama et al., 2021; Tominaga et al., 2021). To the best of our knowledge, no previous study has investigated the relationships between these infections and ustekinumab exposure by using a pharmacovigilance database.

Through data mining, we identified the signal between ustekinumab and possible increased risks of developing HBVr, TB, and AMI in the FAERS pharmacovigilance database. Data mining of pharmacovigilance databases might provide previously unknown or not well-established but clinically important associations (Sakaeda et al., 2013). It is especially more advantageous in the case of rare AEs such as HBVr, TB, and AMI as was the focus of our study. ROR is one of the data mining measures for disproportionality analysis. In our study, it means the odds of reporting HBVr, TB, and AMI with ustekinumab was 2.33, 5.09, and 2.09 times of reporting the AE with other medications use in FAERS, respectively.

Previous evidence is only limited to case reports and series for the association of ustekinumab with HBVr. Eight studies investigating 40 patients with chronic HBV infection receiving ustekinumab uncovered 4 reactivations (10.0%) (Chiu et al., 2013; Navarro et al., 2013; Sanz-Bueno et al., 2015; Raymundo et al., 2016; Almutairi and Abouzaid, 2018; Ting et al., 2018; Piaserico et al., 2019; Siegel et al., 2019). Regarding patients with resolved HBV, three of eighty-five patients (3.53%) treated with ustekinumab from nine studies developed reactivations (Chiu et al., 2013; Koskinas et al., 2013; Hayashi et al., 2014; Steglich et al., 2014; Snast et al., 2017; Almutairi and Abouzaid, 2018; Solay et al., 2018; Ting et al., 2018; Kłujszo et al., 2021). In the current study, we detected a significant signal between ustekinumab exposure and HBVr in FAERS, providing new evidence on their association in terms of pharmacovigilance aspects. Most cases of HBVr with ustekinumab in our study (14/21) and previous studies (6/7) were from Asia (Chiu et al., 2013; Solay et al., 2018; Ting et al., 2018), where HBV infection is endemic. Recently, ustekinumab has been included in medical insurance in mainland China, which holds the largest population of HBV infection worldwide as well as patients that have indications for ustekinumab use. We believe our study will enhance the level of awareness for HBVr when using ustekinumab and improve therapeutic management, especially in this area.

Experience from other immunosuppressive agents showed that the mortality rate of HBVr is about 2% (0–7%) (Lau et al., 2021). The prognosis of HBVr with ustekinumab was generally good and no death was reported in our cohort and previous series (Chiu et al., 2013; Koskinas et al., 2013; Solay et al., 2018; Ting et al., 2018), but one had life-threatening acute liver failure in our cohort. Risk stratification-based management is recommended by guidelines (Reddy et al., 2015; EASL, 2017; Terrault et al., 2018; Lau et al., 2021). Previous eight studies showed that 4 of 40 (10.0%) cases with chronic HBV infection using ustekinumab developed HBVr (Chiu et al., 2013; Navarro et al., 2013; Sanz-Bueno et al., 2015; Raymundo et al., 2016; Almutairi and Abouzaid, 2018; Ting et al., 2018; Piaserico et al., 2019; Siegel et al., 2019), but the 4 did not receive antiviral prophylaxis (Chiu et al., 2013; Ting et al., 2018). Considering about one-quarter of the 40 patients received antiviral prophylaxis, which is highly effective in preventing HBVr, especially with potent nucleos(t)ide analogues (Reddy et al., 2015; EASL, 2017; Terrault et al., 2018; Lau et al., 2021), the anticipated incidence of HBVr would be higher than 10%. This is classified in the high-risk group by the American Gastroenterological Association (AGA) and antiviral prophylaxis is recommended for this group (Reddy et al., 2015). For those with resolved HBV infection, previous nine studies including 85 patients showed that the anticipated incidence of HBVr using ustekinumab was 3.53% (3/85) (Chiu et al., 2013; Koskinas et al., 2013; Hayashi et al., 2014; Steglich et al., 2014; Snast et al., 2017; Almutairi and Abouzaid, 2018; Solay et al., 2018; Ting et al., 2018; Kłujszo et al., 2021), falling within the range of 1%–10%, which is classified in the moderate-risk group by AGA (Reddy et al., 2015). Close monitoring or antiviral prophylaxis is recommended for this group (Reddy et al., 2015; EASL, 2017; Terrault et al., 2018; Lau et al., 2021). Regarding the 85 patients, only 1 received preventive antiviral treatment (Steglich et al., 2014), while the other 84 did not (Chiu et al., 2013; Koskinas et al., 2013; Hayashi et al., 2014; Snast et al., 2017; Almutairi and Abouzaid, 2018; Solay et al., 2018; Ting et al., 2018; Kłujszo et al., 2021). But previous studies as well as the current study were retrospective and/or small sized. Large prospective studies are warranted to obtain a better estimate of the reactivation incidence, so that improved strategies can be established for both HBV carriers and those with resolved HBV infection when using ustekinumab.

Although cases of TB and AMI have been occasionally reported with ustekinumab, (Sánchez-Moya and Daudén, 2012; Tsai et al., 2013; Errichetti and Piccirillo, 2014; Lynch et al., 2017; Shim et al., 2018; Renoux et al., 2020; Tominaga et al., 2021), increased risks to date have not been observed in clinical trials. In fact, ustekinumab is generally safer in terms of infectious complications than TNF inhibitors, the most commonly used biologics in autoimmune diseases (Kalb et al., 2015). A meta-analysis of phase 2 and 3 clinical trials showed only two cases of active TB among 6,581 participants receiving ustekinumab, with a significantly lower rate (0.02 per 100 person-years) compared to that of two representative TNF inhibitors infliximab and golimumab (0.28 per 100 person-years) (Loftus et al., 2017). Even compared with the general population, ustekinumab did not increase the risk of TB, as a national study of 2,803 Korean patients showed (Cho et al., 2020). To the best of our knowledge, this is the first study using pharmacovigilance database to demonstrate the potential risks of developing TB and AMI in ustekinumab-treated patients. In this way by analyzing big data, the risks of rare complications may be detected, while in trials and registry centers they may not. Pre-treatment screening and chemoprophylaxis for latent TB may be another factor that contributes to the inconsistent findings between our study and others. Active TB during immunosuppressive therapy is predominantly from activation of latent TB (Shim, 2014), which can be effectively prevented with isoniazid chemoprophylaxis (Tsai et al., 2012). In clinical trials, patients were screened for latent TB prior to initiating ustekinumab therapy, and those diagnosed with latent TB were either treated with chemoprophylaxis or excluded. Pre-treatment screening and chemoprophylaxis for latent TB are usually well implemented in register centers too. Regarding pharmacovigilance databases, however, they cover heterogeneous populations and in some populations pre-treatment screening and chemoprophylaxis for latent TB are performed at a lower level. In addition, more patients with comorbidities and/or concomitant use of immunosuppressive agents are covered in pharmacovigilance databases and these patients are more likely to develop serious infections.

IL-12 and IL-23 play central roles to regulate T cell-mediated immune responses. In patients with chronic hepatitis B, treatment with recombinant human IL-12 inhibits HBV replication in a dose-dependent manner (Carreño et al., 2000). IL-12 can promote the production of interferon gamma (IFN-γ) by T helper 1 (Th1) cells, while IFN-γ can inhibit HBV replication and induce antiviral effects of HBV-specific cytotoxic T cells (Cavanaugh et al., 1997; McClary et al., 2000; Zeuzem and Carreño, 2001; Guidotti, 2002). Although mechanisms are unknown, a high pre-treatment serum IL-23 level predicts the response to pegylated IFN therapy in patients with chronic hepatitis B (Yu et al., 2015). Patients with inborn errors of IL-12/IL-23 are particularly vulnerable to disseminated infections from tuberculous and nontuberculous mycobacteria, and Bacillus Calmette-Guerin vaccinations (Filipe-Santos et al., 2006). Serious infections and fatal outcomes have been reported in such patients (Filipe-Santos et al., 2006; FDA, 2020). These findings suggest that ustekinumab might theoretically increase the risk of HBVr, TB, and AMI.

The main strength of this study is our ability to detect serious AEs that were not observed during the clinical trial stage for ustekinumab. In clinical trials, participants with chronic hepatitis B or active TB were excluded, or conducted in populations with a low prevalence of exposure to HBV and TB. Sample size of clinical trials is far smaller than real-world data. To the best of our knowledge, this is the largest published series of ustekinumab-associated cases of HBVr, TB, and AMI to date. The data from these cases and significant signals detected in this study suggested caution should be taken with ustekinumab use in susceptible patients and will help prompt utilization of pretreatment screening and on-treatment monitoring and early recognition of these infections in such patients.

Similar to many studies based on pharmacovigilance databases, our study had some limitations. First, due to the voluntary nature of reporting to FAERS, underreporting is expected and reporting bias exists (Goldman, 1998). FAERS allows signal mining for a specific drug and adverse drug reaction of interest but are not enough to establish their relationship. Causality relationship does not necessarily exist even if disproportionality analysis results are significant. Second, incidences and prevalence of HBVr, TB, and AMI cannot be calculated, as the total number of patients using these drugs is undetermined. Third, missing and incomplete information, including medication dosages, timeline to event occurrence, prior use of immunosuppressive agents, baseline HBV status and antiviral prophylaxis for HBVr, and screening and chemoprophylaxis for latent TB were not reported in FAERS. These may act as contributory factors to the development of HBVr, TB, and AMI (Wang et al., 2020). Fourth, the Weber effect and notoriety bias cannot be ruled out in the current study, although studies showed that they are not common in FASRS (Hoffman et al., 2014; Neha et al., 2021).

In summary, we identified positive signals between ustekinumab exposure with HBVr, TB, and AMI in FAERS. Although these complications are rare, clinicians using ustekinumab should be aware of the risks. Clinical trials, pharmacoepidemiological studies, and registries are warranted to confirm the relationship and provide evidence to develop strategies involving pre-treatment screening, monitoring, and utilization of prophylaxis in patients receiving ustekinumab.

## Data Availability

The datasets presented in this study can be found in online repositories. The names of the repository/repositories and accession number(s) can be found below: https://www.fda.gov/drugs/questions-and-answers-fdas-adverse-event-reporting-system-faers/fda-adverse-event-reporting-system-faers-public-dashboard.
