# Supplementary material for: Hepatitis B Virus Reactivation and Mycobacterial Infections Associated With Ustekinumab: A Retrospective Study of an International Pharmacovigilance Database
Source: Front Pharmacol. 2022 Jul 4;13:921084. doi: 10.3389/fphar.2022.921084 (PMC9289361; doi:10.3389/fphar.2022.921084)
Supplement: Supplementary file 1 [file DataSheet3.docx]

**Table S1. Stratified analyses for ROR of HBVr with ustekinumab based on sex, age, reporter, and reporter region in FAERS^#^**

| **Stratum^§^** | **HBVr due to ustekinumab** | **HBVr due to other drugs** | **Other AEs due to ustekinumab** | **Other AEs due to other drugs** | **ROR (95% CI)** |
| --- | --- | --- | --- | --- | --- |
| Sex |  |  |  |  |  |
| Female | 4 | 759 | 26,212 | 9,153,945 | 1.84 (0.69-4.92) |
| Male | 12 | 1,191 | 20,742 | 5,795,858 | 2.82 (1.59-4.97) |
| Age |  |  |  |  |  |
| < 65 years | 13 | 1,094 | 27,318 | 6,406,819 | 2.79 (1.61-4.82) |
| ≥ 65 years | 0 | 694 | 5,845 | 3,745,307 | NA |
| Reporter |  |  |  |  |  |
| Healthcare professional | 16 | 2,535 | 33,567 | 8,415,260 | 1.58 (0.97-2.59) |
| Consumer | 3 | 127 | 16,992 | 8,111,652 | 11.28 (3.59-35.44) |
| Reporter region |  |  |  |  |  |
| US | 4 | 321 | 25,837 | 11,499,210 | 5.55 (2.07-14.87) |
| Other regions | 15 | 2,279 | 24,736 | 5,315,579 | 1.41 (0.85-2.35) |

^#^ Case numbers were based on adverse events reported to FAERS between January 1, 2009 and December 31, 2020.

^§^ Data were available to calculating the ROR stratified by sex, age, reporter, and reporter region through the FAERS dashboard.

AEs: adverse events; CI: confidence interval; FAERS: the United States Food and Drug Administration Adverse Event Reporting System; HBVr: hepatitis B virus reactivation; ROR: reporting odds ratio.

**Table S2. Stratified analyses for ROR of TB with ustekinumab based on sex, age, reporter, and reporter region in FAERS^#^**

| **Stratum^§^** | **TB due to ustekinumab** | **TB due to other drugs** | **Other AEs due to ustekinumab** | **Other AEs due to other drugs** | **ROR (95% CI)** |
| --- | --- | --- | --- | --- | --- |
| Sex |  |  |  |  |  |
| Female | 64 | 5,407 | 26,152 | 9,149,297 | 4.14 (3.24-5.30) |
| Male | 102 | 5,578 | 20,652 | 5,791,471 | 5.13 (4.21-6.24) |
| Age |  |  |  |  |  |
| < 65 years | 69 | 6,805 | 27,262 | 6,401,108 | 2.38 (1.88-3.02) |
| ≥ 65 years | 23 | 2,295 | 5,822 | 3,743,706 | 6.44 (4.27-9.73) |
| Reporter |  |  |  |  |  |
| Healthcare professional | 138 | 9,969 | 33,445 | 8,407,826 | 3.48 (2.94-4.12) |
| Consumer | 62 | 2,637 | 16,933 | 8,109,142 | 11.26 (8.75-14.49) |
| Reporter region |  |  |  |  |  |
| US | 46 | 1,945 | 25,795 | 11,497,586 | 10.54 (7.87-14.13) |
| Other regions | 155 | 10,441 | 24,596 | 5,307,417 | 3.20 (2.73-3.76) |

^#^ Case numbers were based on adverse events reported to FAERS between January 1, 2009 and December 31, 2020.

^§^ Data were available to calculating the ROR stratified by sex, age, reporter, and reporter region through the FAERS dashboard.

AEs: adverse events; CI: confidence interval; FAERS: the United States Food and Drug Administration Adverse Event Reporting System; ROR: reporting odds ratio; TB: tuberculosis.

**Table S3. Stratified analyses for ROR of AMI with ustekinumab based on sex, age, reporter, and reporter region in FAERS^#^**

| **Stratum^§^** | **AMI due to ustekinumab** | **AMI due to other drugs** | **Other AEs due to ustekinumab** | **Other AEs due to other drugs** | **ROR (95% CI)** |
| --- | --- | --- | --- | --- | --- |
| Sex |  |  |  |  |  |
| Female | 6 | 1,270 | 26,210 | 9,153,434 | 1.65 (0.74-3.68) |
| Male | 8 | 1,197 | 20,746 | 5,795,852 | 1.87 (0.93-3.74) |
| Age |  |  |  |  |  |
| < 65 years | 7 | 1,362 | 27,324 | 6,406,551 | 1.21 (0.57-2.53) |
| ≥ 65 years | 4 | 849 | 5,841 | 3,745,152 | 3.02 (1.13-8.07) |
| Reporter |  |  |  |  |  |
| Healthcare professional | 15 | 2,516 | 33,568 | 8,415,279 | 1.49 (0.90-2.48) |
| Consumer | 2 | 357 | 16,993 | 8,111,422 | 2.67 (0.67-10.74) |
| Reporter region |  |  |  |  |  |
| US | 5 | 1,115 | 25,836 | 11,498,416 | 2.00 (0.83-4.80) |
| Other regions | 12 | 1,714 | 24,739 | 5,316,144 | 1.50 (0.85-2.65) |

^#^ Case numbers were based on adverse events reported to FAERS between January 1, 2009 and December 31, 2020.

^§^ Data were available to calculating the ROR stratified by sex, age, reporter, and reporter region through the FAERS dashboard.

AEs: adverse events; AMI: atypical mycobacterial infection; CI: confidence interval; FAERS: the United States Food and Drug Administration Adverse Event Reporting System; ROR: reporting odds ratio.
