# Supplementary material for: Hepatitis B Virus Reactivation and Mycobacterial Infections Associated With Ustekinumab: A Retrospective Study of an International Pharmacovigilance Database
Source: Front Pharmacol. 2022 Jul 4;13:921084. doi: 10.3389/fphar.2022.921084 (PMC9289361; doi:10.3389/fphar.2022.921084)
Supplement: Supplementary file 3 [file DataSheet1.DOCX]

The reporting odds ratio (ROR) can be expressed as

$$ROR=\frac{a/c}{b/d}=\frac{ad}{bc}$$

The 95% confidence interval can be calculated by

$$95\% CI=e^{\ln(ROR)\pm1.96\sqrt{(\frac{1}{a}+\frac{1}{b}+\frac{1}{c}+\frac{1}{d})}}$$

Table A1. Two-by-two contingency table for the ROR calculation

|  | Drug of interest | Other drugs |
| --- | --- | --- |
| Adverse event of interest | a | b |
| Other adverse events | c | d |
